# Feature Selection of OMIC Data by Ensemble Swarm Intelligence Based Approaches

**DOI:** 10.3389/fgene.2021.793629

**Published:** 2022-03-08

**Authors:** Zhaomin Yao, Gancheng Zhu, Jingwei Too, Meiyu Duan, Zhiguo Wang

**Affiliations:** ^1^ Department of Nuclear Medicine, General Hospital of Northern Theater Command, Shenyang, China; ^2^ College of Medicine and Biological Information Engineering, Northeastern University, Shenyang, China; ^3^ Key Laboratory of Symbolic Computation, College of Computer Science and Technology, Knowledge Engineering of Ministry of Education, Jilin University, Changchun, China; ^4^ Faculty of Electrical Engineering, Universiti Teknikal Malaysia Melaka, Hang Tuah Jaya, Melaka, Malaysia

**Keywords:** swarm intelligence (SI), feature selection (FS), transcriptome data, methylation data, intersection and union combination

## Abstract

OMIC datasets have high dimensions, and the connection among OMIC features is very complicated. It is difficult to establish linkages among these features and certain biological traits of significance. The proposed ensemble swarm intelligence-based approaches can identify key biomarkers and reduce feature dimension efficiently. It is an end-to-end method that only relies on the rules of the algorithm itself, without presets such as the number of filtering features. Additionally, this method achieves good classification accuracy without excessive consumption of computing resources.

## 1 Introduction

The OMIC data includes genomes, transcriptomes, metabolomes, and proteomes. (Karczewski and Snyder 2018). Its quantity and quality have been improved significantly during the rapid development and continuous innovation of high-throughput sequencing and mass spectrum technologies (Margolis et al., 2014). Generally, biomedical data has the characteristics of “large p and small n,” that is, the species of features is far larger than the species of samples (Liao and Chin 2007). Thus, it is necessary for biomedical dataset dimension reduction to protect against potential dimension disaster.

Feature selection has been proven with excellent performance in data preprocessing, especially for high dimensional data (Dash and Liu 1997; Bolón-Canedo, Sánchez-Maroño, and Alonso-Betanzos 2015). Its goals consist of cleaning out understandable and analyzable data, constructing simple and efficient models, and improving the efficiency of data mining (Li et al., 2017). It has achieved prominent results in the bioinformation field (Fu et al., 2018; Qiu, Ching, and Zou 2021). Swarm intelligence (SI) is the decentralized self-organizing collective behavior at the collective level (Hu et al., 2021b). It usually consists of a group of simple agents that interact with each other locally and with their environment. The agents follow very simple rules, and there is no centralized control structure to specify the behavior of a single agent. However, the interaction among these agents will lead to the emergence of “intelligent” global behavior (Hu et al., 2021a). Therefore, the whole problem-solving process will not be affected by the failure of one or several agents, so this method has good robustness and potential global search ability. Additionally, SI can transmit and coordinate information through indirect communication. With the increase in the number of individuals, the increase in communication overhead is small. Thus, it also has good scalability. Because of these advantages, SI is widely used in feature selection; its combination with machine learning has especially proven to be able to obtain outstanding results. Through the research and development of the genetic algorithm (Malakar et al., 2019) and the firefly algorithm (Bacanin et al., 2021), the features extracted from each handwritten word image have been significantly optimized so that the performance of the handwritten word recognition technique has been increased visibly.

Various computational feature selection models have been proposed to reduce the dimension of OMIC datasets (Ge et al., 2016; Liu et al., 2017; Yuanyuan, Lan, and Fengfeng 2021). However, these algorithms need to design the number of features in advance as an intervention. Meanwhile, the heuristic rules applied are almost mathematical principles. Thus, this study was intended to investigate the performance of the features screened based on biological or natural rules, instead of traditional mathematical principles, and manually specify the number.

This article is organized as follows: details of the datasets and overview of the methods are described in Section 2. Experimental results and a corresponding analysis of these results are presented in Section 3. Finally, a brief conclusion is drawn in Section 4.

## 2 Materials and Methods

**FIGURE 1:**
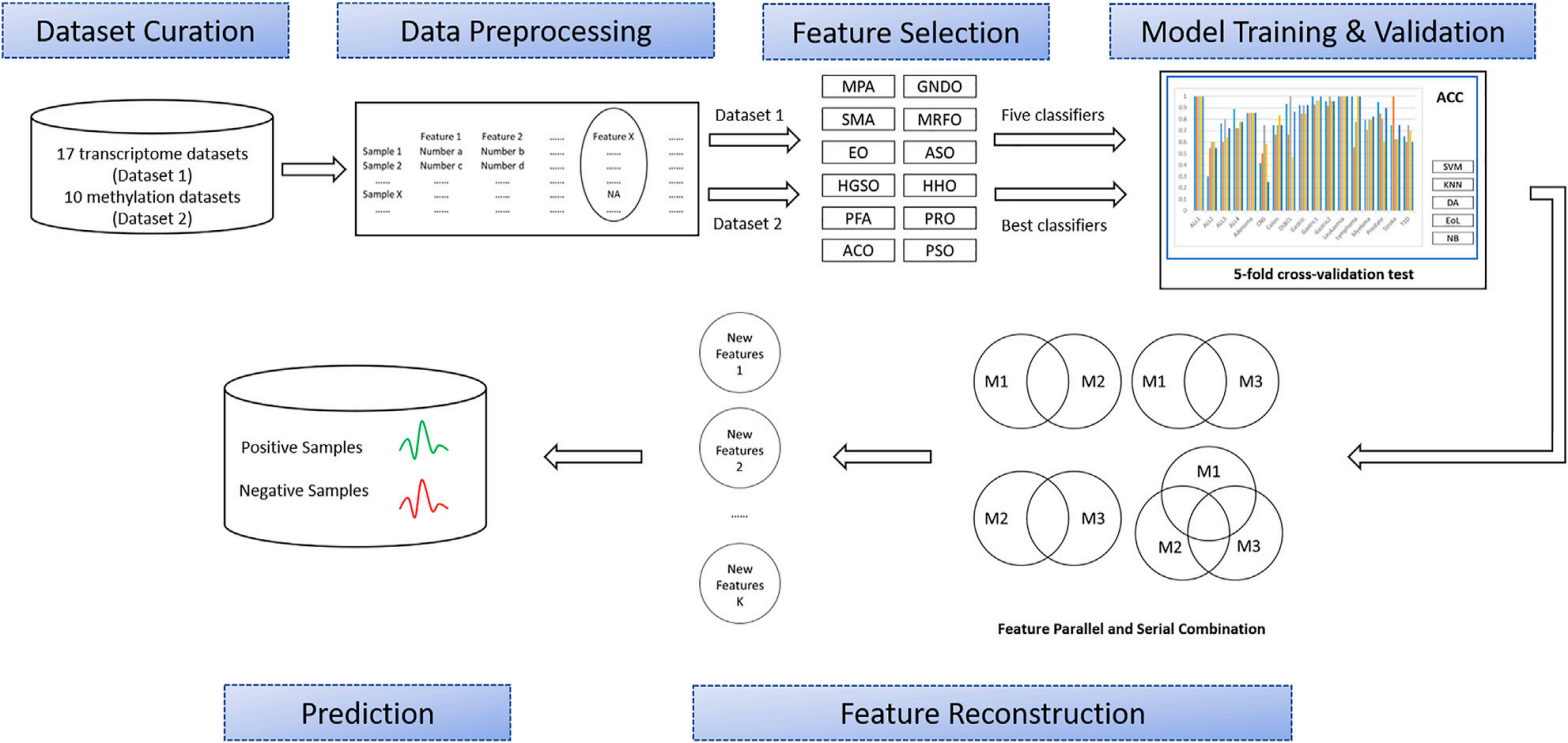
Overview of the proposed methodology.

As shown in Figure 1, this study involved six major stages: Dataset curation, data preprocessing, feature selection, model training and validation, feature intersection and union combination, and prediction. First, a large number of OMIC datasets are collected, including transcriptome datasets (Dataset 1) and methylation datasets (Dataset 2). Then, all the features with missing values in the collected datasets will be deleted. Next, all the transcriptome datasets will have features extracted by twelve advanced swarm intelligent algorithms, and then these features will be input into five different representative classifiers and finally classification performance will be obtained. According to these results, the best classifier and the top three algorithms that use this classifier to get the best results will be selected to apply to methylation datasets. Later, these subsets will generate different combinations through union and intersection. Finally, the classification performance of these combinations will be evaluated by the best classifiers. The details of each process are described in the following sections.

### 2.1 Summary of Datasets

This study concentrated on binary classification and analyzed the relevant publicly available OMIC databases. As shown in Supplementary Table S1, these data include 17 transcriptome datasets and 10 methylation datasets. Methylation is an important modification of proteins and nucleic acids; it reveals the influence of genetic and environmental factors on the occurrence and development of complex diseases (Barros and Offenbacher 2009). Compared with transcriptome data, methylation data usually have more feature dimension and are more challenging in classification.

First, all transcriptome datasets (Dataset 1) were used to test the performance of the algorithm. As shown in Supplementary Table S1, they were DLBCL (Shipp et al., 2002), Pros (Aalinkeel et al., 2004), Colon (Alon et al., 1999), Leuk (Golub et al., 1999), Mye (Tian et al., 2003), All (All1/All2/All3/All4) (Chiaretti et al., 2004), CNS (Pomeroy et al., 2002), Lym (Alizadeh et al., 2000), Adeno (Notterman et al., 2001), Gas (Wu et al., 2013), Gas1/Gas2 (Wang et al., 2013) , T1D (Levy et al., 2012), and Stroke (Krug et al., 2012). These datasets were obtained and preprocessed as similar in Mctwo (Ge et al., 2016).

Additionally, ten methylation datasets (Dataset 2) were used to demonstrate the binary classification performances, as shown in Supplementary Table S1. The dataset GSE74845 profiled 110 Fimbria and 106 proximal tubal DNA samples of fallopian tube fimbriae in BRCA mutation carriers (Bartlett et al., 2016). The dataset GSE80970 provided the methylomes of 148 Alzheimer’s disease samples and 138 controls (Smith et al., 2018). The dataset GSE103186 illustrated 130 gastric light or mild intestinal metaplasia and 61 gastric normal samples (Huang et al., 2017). The dataset GSE139032 investigated 77 lung adenocarcinomas and 77 matched non-malignant lung samples (Enfield et al., 2019). The dataset GSE139404 compared 40 low-grade adenoma and high-grade adenoma in colorectal and 20 normal tissues (Fan et al., 2020). The dataset GSE144910 collected a total of 88 genomic DNA samples taken from the postmortem superior temporal gyrus of the human brain with 44 schizophrenia and paired non-psychiatric controls (Mckinney et al., 2020). The dataset GSE164269 generated 33 discovery and 46 independent validation cohorts of malignant pleural mesothelioma samples (Bertero et al., 2021). The dataset GSE166787 contrasted DNA methylation data throughout human muscle cell differentiation in 28 individuals with type 2 diabetes and 28 controls (Davegårdh et al., 2021). The dataset GSE173330 supplied DNA methylation data from several tissues in toothed whales (*N* = 254) and dolphin (*N* = 291) (Robeck et al., 2021). The last dataset GSE174613 analyzed samples of non-malignancy obtained from prostatectomy specimens (*n* = 12) and of bone metastasis tissue samples obtained from separate prostate cancer patients (*n* = 70) (Ylitalo et al., 2021).

### 2.2 Data Preprocessing

Due to various experimental reasons, gene expression data universally suffer from the missing value problem. The features with missing values can adversely affect the classifiers (Varsha et al., 2016). Considering the number of features with missing values in the datasets accounts for less than 0.1% of the total number of features, direct removal also has little impact on the overall datasets. Thus, these features affected by missing values are removed directly. For example, for a feature *X*, the value of *X* is missing in only one sample, but there is a definite value in all other samples. The X must be removed from all samples.

### 2.3 Summary of Swarm Intelligence Methods in Feature Selection

Twelve swarm intelligence methods are used in the study, including ten state-of-the-art methods from the last 2 years and two classic methods. The methods are briefly described below.

#### 2.3.1 Marine Predators Algorithm

Marine predator algorithm (MPA) is a natural heuristic optimization algorithm. It follows the rule of natural dominance in the optimal foraging strategy and encounters the rate strategy between predator and prey in the marine ecosystem. This algorithm is inspired by the predator–prey strategy in nature and considers that the top predator has the greatest search ability, that is, the decision of a top predator is a solution of the problem (Faramarzi et al., 2020a).

#### 2.3.2 Generalized Normal Distribution Optimization

Generalized normal distribution optimization (GNDO) is a novel metaheuristic algorithm inspired by normal distribution theory. It can solve optimization problems by natural phenomenon distribution and fitting minimum standard variance of the positions of all individuals. Generally speaking, GNDO consists of two main strategies: local exploitation and global exploration. The former focuses on building the generalized distribution model while the latter explores the search region based on three randomly selected individuals (Zhang et al., 2020).

#### 2.3.3 Slime Mould Algorithm

Slime mould algorithm (SMA) is based on the diffusion and foraging behavior of slime mould in nature. It calculates the optimal path by simulating the relationship between morphological changes and contraction patterns of slime mould during foraging. SMA performs the search relying on three stages: Find approach, wrap food, and oscillation (Li et al., 2020).

#### 2.3.4 Manta Ray Foraging Optimization

Manta ray foraging optimization (MRFO) mathematically models and mimics three unique foraging strategies of manta rays, including chain foraging, cyclone foraging, and somersault foraging, for solving global optimization problems. In chain foraging, the manta rays update their solutions by following the best solution and the solution in front of it. For cyclone foraging, the manta rays move toward the global optima along a spiral path. Last, in somersault foraging, the manta rays tend to update their position around the best solution in the population (Zhao et al., 2020).

#### 2.3.5 Equilibrium Optimizer

Equilibrium optimizer (EO) is inspired by a physical phenomenon of controlling volume mass balance. It simulates the physical process of mass entering, leaving, and generating in the control volume to finally reach the equilibrium state as optimal results. In EO, there is an equilibrium pool that used to store the current four best-so-far solutions. Iteratively, these stored solutions will be applied to enhance the quality of solutions in the population. Additionally, EO integrates the particle memory saving to benefit the exploitation capability (Faramarzi et al., 2020b).

#### 2.3.6 Atom Search Optimization

Atom search optimization (ASO) is a novel algorithm based on a basic molecular dynamics model. In a molecular system, there are interaction forces between neighboring atoms, and the globally optimal atoms constrain other atoms. Gravitation makes atoms explore the whole search space extensively, and repulsion makes them develop the potential region effectively. It simulates this phenomenon to find the global optimal solution (Zhao et al., 2019).

#### 2.3.7 Henry Gas Solubility Optimization

Henry gas solubility optimization (HGSO) is a novel metaheuristic algorithm; it imitates the huddling behavior of gas described in Henry’s law to balance the exploitation ability and the exploration ability of the algorithm for searching the global optimum and avoid trapping into local optima (Hashim et al., 2019).

#### 2.3.8 Harris Hawks Optimization

Harris hawks optimization (HHO) is a novel population-based, natural heuristic optimization. Its main inspiration comes from Harris’s eagle’s cooperative behavior and pursuit in nature. It is unique because it has a unique cooperative foraging activity with other family members in the group. Because of this, it is very suitable to simulate the unique predatory behavior of Harris’s hawk as a swarm intelligence optimization process (Heidari et al., 2019).

#### 2.3.9 Path Finder Algorithm

Path finder algorithm (PFA) is inspired by the hunting behavior of group animals. The algorithm realizes the optimization process through the communication between pathfinder and follower from the population in the process of the population searching for food. Naturally, PFA stores the best-so-far solution (pathfinder), in which the pathfinder is used to enhance the exploitation and exploration capability (Yapici and Cetinkaya 2019).

#### 2.3.10 Poor and Rich Optimization

Poor and rich optimization (PRO) is developed based on the real social phenomenon, that is, the attempt of the rich and the poor to improve their economic conditions. This social behavior can be regarded as a solution for complex optimization problems. In PRO, a mutation operator is designed to improve the compound population. Even though PRO is a promising algorithm, it suffers from the high computational complexity (Moosavi and Bardsiri 2019).

#### 2.3.11 Ant Colony Optimization

Ant colony algorithm is inspired by the foraging behavior of ants in nature. In the process of ant foraging, an ant colony can always find an optimal path between the ant nest and food source. This is because the ants in the ant colony can transmit information through some information mechanism. After further research, it is found that ants will release a substance called “pheromone” on their path. Ants in the ant colony have the ability to perceive the “pheromone.” They will walk along the path with high concentration of “pheromone,” and each passing ant will leave “pheromone” on the road, which forms a mechanism similar to positive feedback; in this way, after a period of time, the whole ant colony will reach the food source along the shortest path (Dorigo et al., 2006).

#### 2.3.12 Particle Swarm Optimization

Particle swarm optimization is inspired by the study of bird predation behavior. Specifically, birds find the optimal destination through collective information sharing. In PSO, the potential solution of each optimization problem is a bird in the search space, which is called a particle. All particles have a fitness value determined by the optimized function, and each particle also has a speed to determine their flying direction and distance. Then the particles follow the current optimal particle to search in the solution space (Kennedy and Eberhart 1995).

### 2.4 Model Training and Validation

#### 2.4.1 Random 5-Fold Cross-Validation Strategy

K-fold cross-validation is one of the most commonly used evaluation strategies. This experimental procedure is performed by the 5-fold cross-validation, that is, the baseline dataset is randomly divided into five equal parts (the number and distribution of samples are the same) and the test processes are repeated five times; for each cross-validation test, one subset is used for testing while the remains are used for training the model. The final performance is represented by the average of five experimental results.

#### 2.4.2 Leave-One-Out Cross-Validation Strategy

Leave one method cross-validation is to treat each data sample as an independent dataset, use one sample each time as the test set, and use all the remaining samples as the training set. The result obtained using this method is closest to the expected value of the whole test set, but the computing cost is excessively expensive.

#### 2.4.3 Performance Evaluation of Various Classifiers

Higher classification accuracy and fewer features are the objectives of generating models; however, it is difficult to achieve both at the same time. Here, the first consideration in this study is the classification accuracy. For achieving a more comprehensive and stable performance, five widely used classifiers are applied to the models, that is, support vector machine (SVM), K-Nearest Neighbor (KNN), discriminant analysis (DA), ensemble of learners (EoL), and naive Bayes (NB). This study evaluates a feature subset through the best classification performance of multiple classifiers. Generally, prediction accuracy is defined as follows:

$$ACC=\frac{TP+TN}{TP+FP+TN+FN}$$

where TP, FP, TN, and FN represent the value of true positives, false positives, true negatives, and false negatives, respectively.

### 2.5 Feature Intersection and Union Combination

**FIGURE 2:**
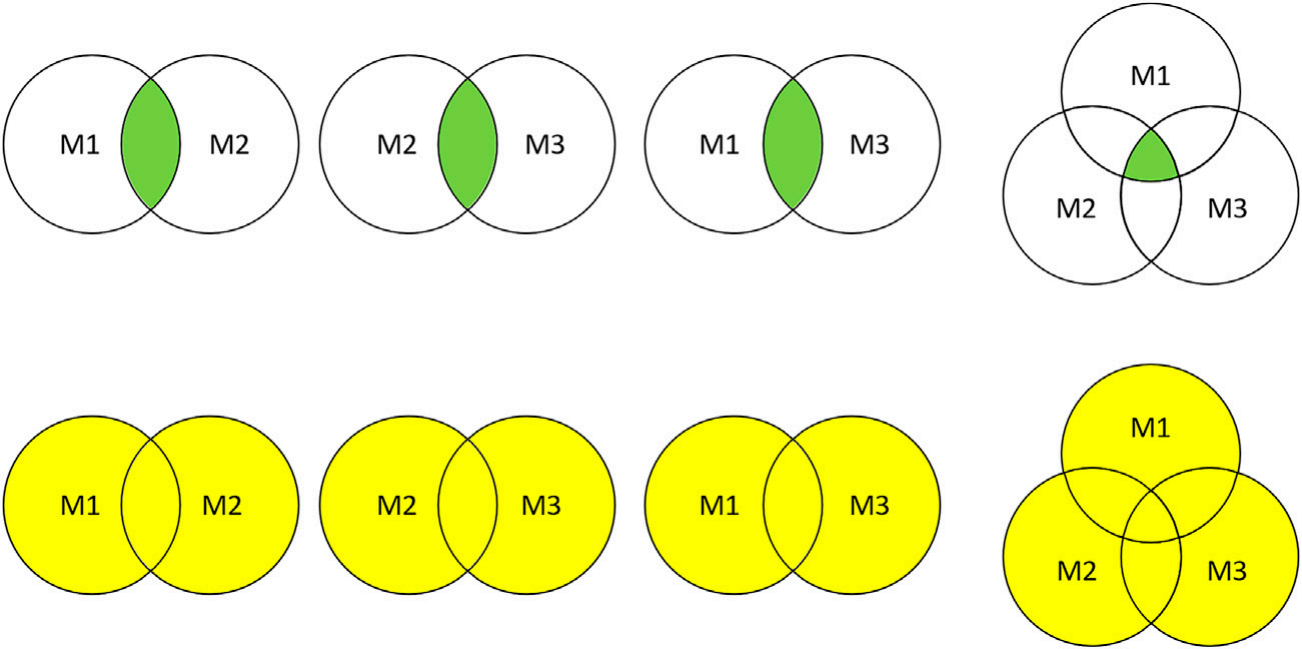
Feature subsets combination. M1, M2, and M3 represent the feature subsets extracted by three different methods, respectively. The green part and yellow part represent the combination results obtained by intersection and union.

Intersection and union combination approaches were employed to ensemble the selected features. As shown in Figure 2, two or three different feature selection results were combined into eight subsets for performance comparison.

## 3 Results and Discussions

### 3.1 The Result on Transcriptome Datasets

This study used these transcriptome datasets for testing the performance of baseline swarm intelligence algorithms and classifiers. Enough iterations are used to satisfy the fitness value. Here, the random 5-fold cross-validation and leave-one-out cross-validation are used to evaluate the performance, respectively. The results are shown in Supplementary Tables S2, S3. Both of the tables show that KNN can make most datasets achieve the best classification effect in most algorithms. Additionally, in the other three algorithms, where KNN cannot achieve the best results, the gap between KNN and the best classifier in the number of datasets for best performance is small, only one to three datasets.

Through the information combination of two tables, when using KNN, the number of best results obtained by PFA and SMA is 12 and 8, respectively, ranking first and second. ASO, GNDO, PSO, and HGSO all get 7 best results, and the number is equal. As shown in Supplementary Table S4, considering the average number of features used on each dataset, HGSO is chosen as the last algorithm to be applied to the next stage.

Because there is little performance difference between 5-fold cross-validation and leave-one-out cross-validation in these transcriptome datasets and the computing cost of leave-one-out cross-validation is relatively high, the subsequent evaluation is only based on the random 5-fold cross-validation.

### 3.2 Convergence of Top Three Swarm Intelligence Algorithms

In the FS phase, a fitness function is adopted to evaluate the quality of the initial and newly generated solutions. This study evaluates the solutions by considering the minimum classification error and minimum size of features (Emary et al., 2016a). Mathematically, the fitness function is defined as follows:

$$Fit=\beta ER+\left(1-\beta \right)\left(\frac{\left|SF\right|}{\left|AF\right|}\right)$$

where *ER* is the classification error rate computed by the *k*-nearest neighbor classifier (KNN, *k*-value = 5), |*SF*| is the number of the selected features, |*AF*| is the total number of features, and *β* is the weight factor between 0 and 1. This study adopts *β* = 0.99 since the classification performance is the most importance measurement (Emary, Zawbaa, and Hassanien 2016b; Mafarja et al., 2019). In the fitness evaluation stage, the dataset is partitioned into training and validation sets using the *k*-fold cross-validation method. Consequently, the dataset is divided into 5 folds, in which *k*-1 folds are used to build the training set while the rest is kept for accessing the selected features.

**FIGURE 3:**
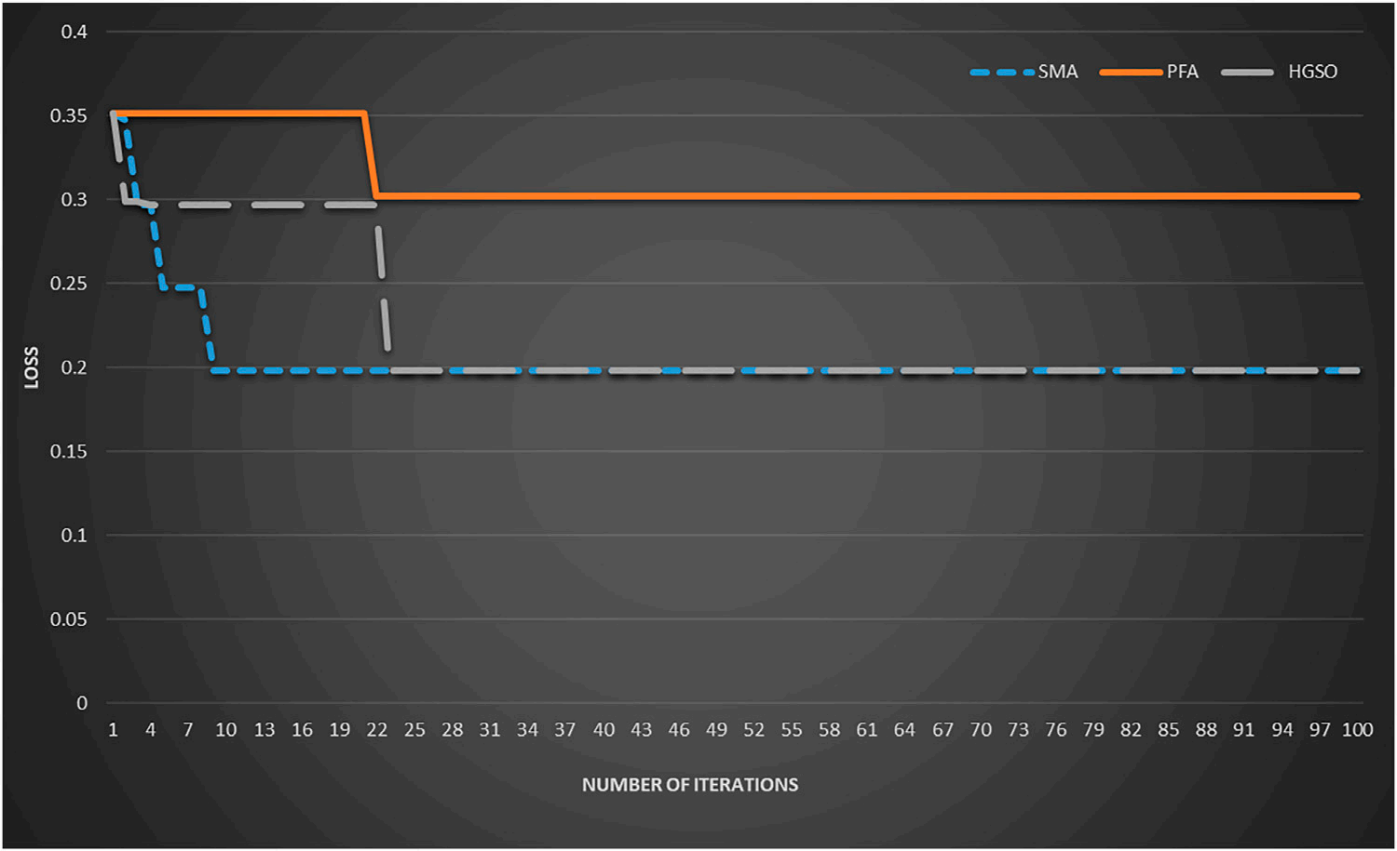
The convergence speed of top three swarm intelligence algorithms on T1D.

The T1D dataset is used as an example to show the convergence of the top three algorithms. As shown in Figure 3, PFA and HGSO converge in about 22 iterations, while SMA converges faster, and the convergence can be completed in about 10 iterations.

### 3.3 The Result of Top Three Swarm Intelligence Algorithms on Methylation Datasets

This section evaluated the performance of SMA, PFA, and HGSO on the methylation datasets, and the classifier is KNN.

**FIGURE 4:**
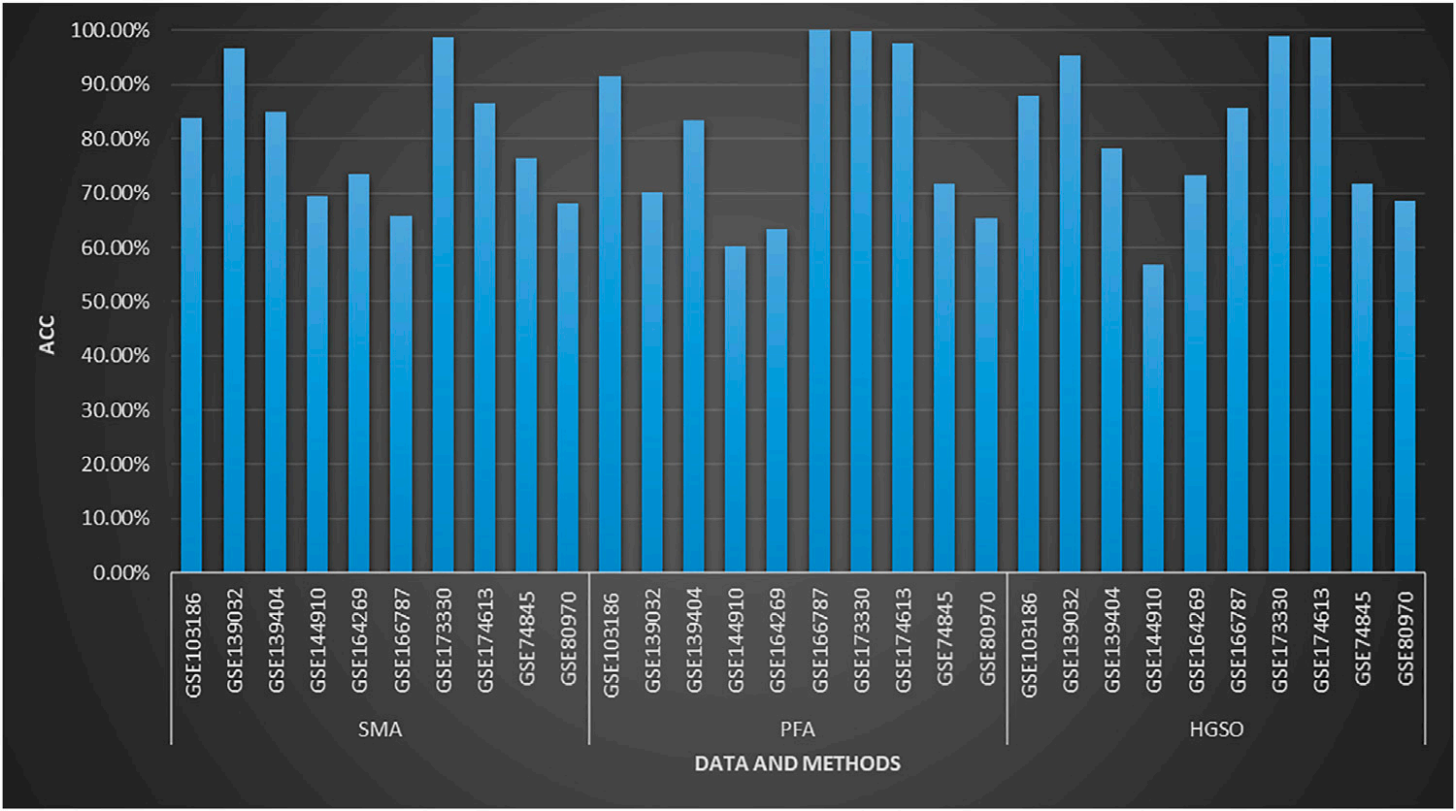
Performance of three swarm intelligence algorithms on methylation datasets.

Although methylome datasets may be a challenge for many feature selection algorithms, the swarm intelligence algorithm has achieved good results on many datasets. As shown in Figure 4, PFA achieves more than 90% accuracy on four datasets. Meanwhile, SMA obtains about 90% accuracy on the GSE139032 and GSE139404, where PFA does not get good results. In addition, the consumption of computing resources and time is also within an acceptable range; the average time consumption (CPU: i9-11900H) of SMA, PFA, and HGSO are 101.83, 415.21, and 312.31 s, respectively.

### 3.4 Other Evaluation Indexes of Top Three Swarm Intelligence Algorithms on Methylation Datasets

Besides accuracy, other evaluation indicators are also very important. They can reveal the characteristics of the algorithm in other aspects. Therefore, another four commonly used indicators for classification evaluation (precision, recall, F1-score, and AUC ROC) have also been tested, and the results are shown in the Supplementary Table S5. It can be seen from the results that there is little difference between precision and recall of most models. However, the precision of PFA reaches 100% but the corresponding recall just obtains about 12% on GSE164269. It may be caused by the insensitivity of the dataset to the algorithm, that is, the algorithm cannot filter the core features of the dataset. Thus, many positive samples are identified as negative samples.

### 3.5 Statistical Tests of Obtained Results

Statistical tests on the results obtained using the three methods were performed. The statistics are described in Table 1. The result of Wilcoxon signed ranks test are shown in Table 2. Through the nonparametric test of paired samples, the *p*-values are greater than the significance level, indicating that there is no difference in the measurement accuracy of these 10 samples after three methods. Additionally, the Friedman test was also applied, and the chi-squared, df, and *p*-value are 0.2, 2, and 0.906, respectively. It also proved that there was no significant difference in accuracy.

**TABLE 1:** Descriptive statistics of the results on methylation datasets.

Methods	Sample number	Average (%)	Standard deviation	Min (%)	Max (%)
SMA	10	80.44	11.62	65.91	98.72
PFA	10	80.30	15.98	60.13	100.00
HGSO	10	81.55	14.17	56.73	98.90

**TABLE 2:** Wilcoxon signed ranks test.

Comparison	*R* ^*+*^	*R* ^*−*^	*p*-value
PFA versus SMA	4	6	0.721
HGSO versus SMA	5	5	0.959
HGSO versus PFA	5	5	0.878

### 3.6 The Result of Feature Intersection and Union Combination on Methylation Datasets

Generally, for a given dataset, the feature subsets for different feature selection are individually somewhat different due to the different theories. So, their different combinations will be more diverse. These subsets are evaluated in this section. What is more, there is no duplicate selection of the same features by different methods.

**FIGURE 5:**
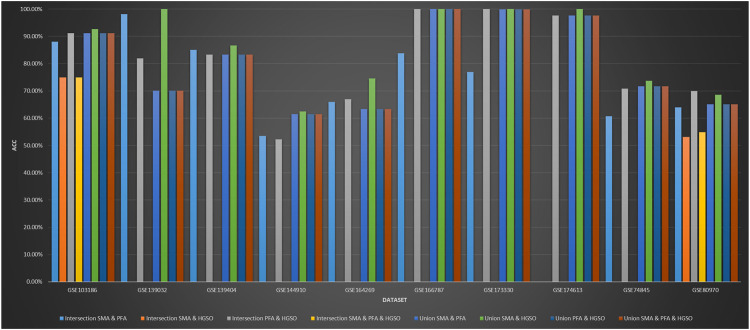
Performance of feature intersection and union combination on methylation datasets.

Figure 5 shows the classification performance obtained by intersection and union combination-based feature subset ensemble methods. In some feature subset combinations, no classification accuracy is available because there is no repeat selection of the same features by the applied methods. As we can see, the performance of the union combination method with PFA is not obvious. The reason may be that PFA selects too many features, which is over 2000 times that of SMA and about 200 times that of HGSO. Additionally, the performance of union combination between SMA and HGSO is always better than just using HGSO but not always better than just using SMA. The reason may be that the number of features used by HGSO is ten times than that of SMA. Therefore, the characteristics of SMA can only be used for auxiliary adjustment. What is more, the performance of some intersection methods does not decrease so much. This may be because the features selected by all these algorithms are the core features of the datasets.

### 3.7 The Feature Selection Rates on Methylation Datasets

Table 3 shows the feature selection rates of single and different combination swarm intelligence methods on methylation datasets. Note that the feature selection rate is the percentage of the features that are extracted from the original features.

**TABLE 3:** Feature selection rates of all used feature subsets on methylation datasets.

Data	Solo			Intersection				Union			
	SMA (%)	PFA (%)	HGSO (%)	SMA and PFA	SMA and HGSO	PFA and HGSO (%)	SMA and PFA and HGSO	SMA and PFA (%)	SMA and HGSO (%)	PFA and HGSO (%)	SMA and PFA and HGSO (%)
GSE103186	0.0338	49.7048	0.6381	0.0154%	0.0002%	0.3184	0.0002%	49.7232	0.6716	50.0245	50.0428
GSE139032	0.0218	49.8948	0.0181	0.0145%	—	0.0109	—	49.9021	0.0399	49.9021	49.9093
GSE139404	0.0009	49.7509	0.0328	0.0004%	—	0.0149	—	49.7513	0.0336	49.7688	49.7692
GSE144910	0.0004	49.9814	0.0046	0.0001%	—	0.0018	—	49.9816	0.0049	49.9841	49.9844
GSE164269	0.0044	49.9655	0.7131	0.0022%	—	0.3630	—	49.9677	0.7175	50.3156	50.3178
GSE166787	0.0017	49.6841	0.0111	0.0009%	—	0.0059	—	49.6849	0.0129	49.6893	49.6902
GSE173330	0.0160	48.7964	0.3728	0.0107%	—	0.1651	—	48.8017	0.3888	49.0041	49.0094
GSE174613	0.0008	49.4005	0.0066	—	—	0.0049	—	49.4014	0.0074	49.4022	49.4030
GSE74845	0.0023	49.9412	0.1849	0.0011%	—	0.0933	—	49.9425	0.1873	50.0328	50.0341
GSE80970	0.1564	49.9624	0.6070	0.0871%	0.0007%	0.3080	0.0005%	50.0317	0.7626	50.2614	50.3304
Average	0.0238	49.7082	0.2589	0.0147%	0.0005%	0.1286	0.0004%	49.7188	0.2827	49.8385	49.8491

As we can see, SMA produces the lowest feature reduction rate in a single model, that is, the average is 0.0238%. This means that applying SMA as the embedded feature selection method may cause “over selection,” with too many informative features filtered out. On the other hand, PFA not only allows selection of the most informative features but also avoids the risk of over selection. However, using the intersection combination with HGSO and PFA not only can reduce the number of features further but also not reduce the accuracy in many datasets. The results indicate that intersection combination method-based ensemble feature selection is likely to play a positive role in filtering out information redundancy among the feature selection methods that retain too much information after use.

In addition, using the combination among feature subsets with widely different feature numbers will not lead to excessive changes in classification performance, and most of the classification results will be the result of the feature subset with the highest number of features, because its feature distribution has not changed.

### 3.8 The Results of Multi-Classification on GSE103186

The internal metaplasia samples contained in GSE103186 can also be more finely divided into classic and mild. Therefore, GSE103186 is regarded as a three-category dataset for testing the multi-classification performance. The performance of SMA, PFA, and HGSO is 81.69, 80.63, and 83.78%, respectively. Although the proposed method mainly focuses on binary classification problems, the results show that it still has the potential to be used in multi-classification problems.

### 3.9 Biological Function Analysis of Selected Features on GSE144910

**FIGURE 6:**
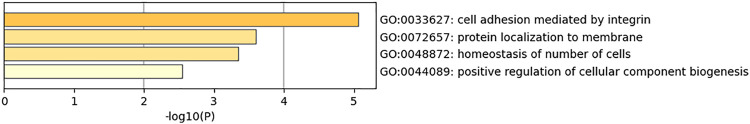
Performance of feature intersection and union combination on methylation datasets.

The dataset GSE144910 collected DNA samples from the superior temporal gyrus of the human brain for researching schizophrenia. The features detected by the union combination of SMA and HGSO as the classification biomarkers and these methylation features are related to 18 genes, which are C1orf168, CAMLG, SMOX, KCNIP4, MIR658, CENPA, ASRGL1, PISD, HNRNPL, EEF2K, GMDS, MPPED1, ANKRD54, PLEK2, ADA, RNF121, KRT6A, and EPHA2. In order to explore the biological functions of the selected genes, pathway analysis was conducted. Figure 6 showed the mainly obtained four biological process pathways (GO: 0033627, 072657, 00488872, and 0044089). We found that schizophrenia may be related to the function of cell adhesion.

## 4 Conclusion

This study focuses on examining the binary classification performance of swarm intelligence algorithms on OMIC datasets. The experimental results suggest that swarm intelligence algorithms can achieve high accuracy on the collected OMIC datasets, significantly reduce feature dimensions, and identify key features. Meanwhile, this study finds some rules to improve ensemble feature subset performance through intersection and union combination methods. However, there are still some limitations in the proposed study. For example, the methodology framework has not been improved, and there is no methodological fusion of different swarm intelligence algorithms. Our future research will focus on combining machine learning and swarm intelligence approaches for reducing the feature dimension and improve the accuracy further in OMIC data and other biological data.

## Data Availability

Publicly available datasets were analyzed in this study. The data can be found at: http://www.broadinstitute.org/cgi-bin/cancer/datasets.cgi (Broad Institute Genome Data Analysis Center) and https://www.ncbi.nlm.nih.gov/geo/ [NCBI Gene Expression Omnibus (GEO) database].
